# Inaccurate communication in health sciences: The case of ‘partial artemisinin resistance’ for the treatment of malaria

**DOI:** 10.1016/j.nmni.2024.101544

**Published:** 2024-11-30

**Authors:** T. Hanscheid, Sara M. Mahomed, Maria Rebelo, Susana Oliveira Henriques, Martin P. Grobusch

**Affiliations:** a Faculdade de Medicina, Universidade de Lisboa, Lisboa, Portugal; b Gulbenkian Institute for Molecular Medicine, Lisboa, Portugal; c Serviço de Doenças Infeciosas, Unidade Local de Saúde de Santa Maria, Lisboa, Portugal; d Center of Tropical Medicine and Travel Medicine, Department of Infectious Diseases, Amsterdam University Medical Centers, Location AMC, Amsterdam Infection & Immunity, Amsterdam Public Health, University of Amsterdam, Amsterdam, the Netherlands

**Keywords:** Partial artemisinin resistance, Malaria, Scientific communication, PubMed, Terminology, GPT-4, Corpus analysis

## Abstract

**Background:**

Accurate scientific terminology is crucial in health sciences to avoid misinterpretations. The use of ‘artemisinin resistance’ to describe delayed parasite clearance may be inaccurately equated with full resistance, as is typically the case when ‘resistance’ is used with other pathogens, leading to potential confusion. In 2018, the World Health Organization (WHO) introduced ‘partial artemisinin resistance’ to more accurately reflect the delayed parasite clearance observed with artemisinin-based therapies.

**Methods:**

We analyzed whether articles in PubMed accurately convey the concept of ‘partial artemisinin resistance’ using GPT-4 to generate related search terms. AntConc was employed for corpus analysis of retrieved articles to examine terminology in titles and abstracts. A manual review evaluated the use of the WHO concept of ‘partial artemisinin resistance’ in the full text of a subset of high-impact articles.

**Results:**

Out of 4041 articles retrieved, only 7 % (n = 281) used ‘partial’ or ‘delayed’ in titles or abstracts. Even after 2018, when WHO introduced the term ‘partial artemisinin resistance’, only 10 % of articles included this. Manual analysis of 161 full-text articles revealed that 94 % did not use ‘partial artemisinin resistance’, and 59 % did not explain the concept of delayed parasite clearance.

**Conclusion:**

The delayed introduction of the term ‘partial artemisinin resistance’ may have contributed to continued use of the scientifically questionable term ‘artemisinin resistance’. This term may be misunderstood as full resistance, as is common with antibiotic resistance. Accurate terminology is essential for clear scientific communication, and precise terms should be established and consistently used from the outset by scientists and clinicians.

## Highlights

• Accurate communication in health sciences is crucial to prevent errors.
• Partial artemisinin resistance involves delayed clearance, but not full resistance.
• Despite WHO's introduction of the term, only 10 % of articles post-2018 included it.
• This study underscores need for precise terms to avoid misinterpretations.

## 1 Introduction

Effective communication and precise terminology are essential in science, particularly in health sciences, where misunderstandings could have serious consequences. In infectious diseases and microbiology, ‘resistance’ is defined as the ability of microorganisms to withstand the effects of a drug that once could successfully eliminate them (Table 1) [[1], [2], [3]]. In clinical practice, ‘resistance’ implies that a microorganism will not respond to standard treatment, necessitating alternative medications [4,5]. For instance, incorrect treatment of a resistant infection can lead to treatment failure and further resistance. *Escherichia coli* can develop resistance to antibiotics like ampicillin, which forces clinicians to choose an alternative treatment [6]. Similarly, *Mycobacterium tuberculosis* can develop resistance to key drugs such as isoniazid and rifampicin, excluding these drugs from the treatment of tuberculosis [7]. The same clinical interpretation of the term resistance usually also applies to antimalarial drugs (Table 1) and combinations such as chloroquine [8,9], sulfadoxine-pyrimethamine [10], or mefloquine [11], where resistance means the drug is ineffective, requiring the use of alternative medications or combinations [12,13].

**Table 1:** Definitions of resistance by EUCAST and WHO.

Category	Definition
EUCAST (2024)^a^	
S - Susceptible, standard dosing regimen	A microorganism is categorised as “Susceptible, standard dosing regimen” when there is a high likelihood of therapeutic success using a standard dosing regimen of the agent.
I - Susceptible, increased exposure∗	A microorganism is categorised as “Susceptible, Increased exposure∗" when there is a high likelihood of therapeutic success because exposure to the agent is increased by adjusting the dosing regimen or by its concentration at the site of infection.
R - Resistant	A microorganism is categorised as “Resistant” when there is a high likelihood of therapeutic failure even when there is increased exposure.
**WHO (2022)**^b^	
Antimalarial drug resistance	Defined as the ability of a parasite strain to survive and/or multiply despite the administration and absorption of a drug given in doses equal to or higher than those usually recommended, but within tolerance of the subject
Artemisinin partial resistance	Defined as delayed clearance after treatment with a drug containing an artemisinin derivative of a parasite strain carrying a particular mutation or set of mutations that are validated as associated with this delayed clearance, despite the administration and absorption of the drug given in doses equal to or higher than those usually recommended, but within tolerance of the subject.

a EUCAST redefined S, I, and R in 2019 for clarity. The new “I" highlights susceptibility with increased exposure, while “Resistant” (R) unequivocally indicates a high likelihood of therapeutic failure, even with increased exposure [2].

b WHO document refers to this: Treatment failure is distinct and can result from various factors beyond resistance (e.g., poor compliance or drug quality). Artemisinin partial resistance, introduced by WHO in 2018 as “tolerance,” refers to delayed parasite clearance linked to PfKelch13 mutations. While “tolerance” is scientifically accurate, “partial resistance” is preferred for broader communication to non-expert audiences [3].

Artemisinin and Artemisinin-based Combination Therapies (ACTs) have shown effective treatment responses by rapidly killing malaria parasites [14]. Since 2001, ACTs have been recommended as the first-line treatment for malaria [13], and in 2010, intravenous artesunate became the first-line treatment for severe *Plasmodium falciparum* malaria [15]. However, in 2008, reports from Western Cambodia highlighted instances of ‘delayed parasite clearance’ after treatment with artesunate monotherapy or artesunate-mefloquine [16,17], characterized by detectable parasitemia on day 3 (72 h) post-treatment [17]. This phenomenon was initially termed ‘artemisinin resistance’ and was characterized by longer time for parasites to be cleared from the patient's bloodstream, despite the treatment remaining clinically effective [17]. Notably, in vitro studies indicated that delayed parasite clearance is associated with changes in ring-stage survival assays rather than overall shifts in IC50 values on conventional assays [3,16]. While defining ‘true’ delayed parasite clearance remains highly complex [18,19], the definition applied in the abovementioned paper [17] serves as an acceptable proxy for this context.

A decade later, in 2018, the World Health Organization (WHO) introduced the term ‘partial artemisinin resistance’ to emphasize that delayed clearance is distinct from full resistance and does not necessarily predict treatment failure for ACTs (Table 1) [20,21]. WHO explicitly stated that “using the term artemisinin resistance is not accurate based on currently available data” [3]. Instead, they recommend using the terminology ‘partial resistance’ or ‘tolerance’ to improve messaging and avoid conflating delayed clearance with treatment failure [3]. This distinction ensures the accurate communication of artemisinin's continued efficacy unless partner drug resistance is present.

Despite recent publications highlighting the implications of only using the term ‘artemisinin resistance’ [[21], [22], [23], [24]], it remains widely used as evidenced by a recent publication in a journal from the American Society for Microbiology [25], known for their advocacy of precise scientific language. Misinterpretations arise from equating ‘artemisinin resistance’ with the concept of traditional ‘antimicrobial resistance’. For instance, a ProMED post cited an article from *The Guardian* that alarmingly referred to ‘aggressive multi-drug-resistant malaria’, suggesting that malaria is now untreatable with the best drugs, seemingly including artemisinin [26]. Without using or explaining ‘partial artemisinin resistance’, policymakers or clinicians unfamiliar with this concept may mistakenly believe that artemisinin is clinically ineffective due to complete ‘resistance’. This misunderstanding could result in clinicians choosing alternative treatments that may lead to poorer outcomes, such as using quinine over intravenous artesunate for a patient with severe malaria, mistakenly believing it to be a more reliable option, even though it is less effective [27,28].

Typically, scientific information flows from original research to reviews and opinion pieces (such as editorials) in scientific publications [29]. From these sources, information disseminates to search engines like Google, semi-scientific entities such as government sites and internet platforms, and popular science publications like *Scientific American*, eventually reaching the mainstream media [30,31]. In this hierarchical flow of information, peer-reviewed scientific journals listed in PubMed are usually considered the most reliable sources of accurate scientific knowledge and precise terminology.

The objective of this study was to analyze whether articles indexed in the PubMed and Web of Science (WoS) databases accurately convey the concept of artemisinin resistance by either using the correct term ‘partial artemisinin resistance’ or clearly explaining it as delayed parasite clearance.

## 2 Methods

### 2.1 PubMed search for ‘artemisinin resistance’

The search process began with the general term ‘artemisinin resistance’ across all fields, followed by focused searches in title/abstract [tiab] fields and using Medical Subject Headings [MeSH] terms (Suppl. Table 1). The MeSH terms used were ‘artemisinin’ and ‘drug resistance’. GPT-4 was used to create relevant search terms related to all possible variants of the words ‘artemisinin’ AND ‘resistance’ in [tiab] fields, also incorporating ‘partial’ OR ‘delayed’ to assess the use of the WHO concept of partial artemisinin resistance related to delayed parasite clearance (Suppl. Table 2). AntConc (version 4.2.4, released on September 25, 2023) was used to conduct a corpus analysis of the extracted titles and abstracts from the retrieved hits, allowing for a detailed examination of the terminology used in the literature.

### 2.2 Web of Science (WoS) search and cross-referencing with PubMed search

The search was restricted to publications from 2018 onward, as this marked the publication of the first WHO definition of ‘partial artemisinin resistance’ [20]. Unlike PubMed, WoS allows extracting additional information for each hit in CSV format. A WoS search was conducted to further characterize the subset of publications and to identify those specifically addressing artemisinin resistance, without focusing on studies mentioning partial or delayed resistance in [tiab]. Results were filtered to include publications since 2018 with a Digital Object Identifier (DOI) and more than 10 citations, to focus on more widely recognized studies. Data were cross-referenced with the PubMed subset ‘J,’ which includes publications containing all variants of ‘artemisinin’ AND ‘resistance’ in [tiab] OR [MeSH] terms, excluding entries mentioning ‘partial’ or ‘delayed’. All data were exported into Excel (version 16.86, Microsoft 365 Subscription). AntConc (version 4.2.4, released on September 25, 2023) was used to perform a corpus analysis for the extracted titles and abstracts from the filtered hits, facilitating a detailed examination of the terminology used in the literature and confirming the absence of ‘partial’ or ‘delayed’ (Suppl. Table 1). This identified recent, well-cited publications that likely do not effectively convey the WHO concept of ‘partial artemisinin resistance’.

### 2.3 Manual verification of selected full-text articles

To confirm accurate communication of ‘partial artemisinin resistance’, selected WoS articles with more than 10 citations were reviewed. The selection included: a) all publications with more than 50 citations, and b) a random sample of 150 publications with more than ten but fewer than 50 citations. These documents were converted into a single merged TXT file for corpus analysis using AntConc to search for terms ‘resistance’ and ‘artemisinin resistance’, along with ‘partial’ and ‘delayed’ to assess the WHO concept of ‘partial artemisinin resistance’.

Manual evaluation process of full-text articles included the following criteria:

a)Classification of publication: each publication was categorised into basic research, epidemiological, or clinical studies;

b)Likelihood of general interest: rated on a scale of 1–3: (1) very likely: articles such as trials on drug efficacy or clinically relevant epidemiology data, (2) perhaps likely: articles covering sub-aspects like detection methods of partial resistance or clinically relevant comparisons with other drugs, (3) unlikely: highly specialized molecular or genetic details, or where artemisinin resistance was only relevant in a remote context of the paper;

c)The transmission of the partial artemisinin resistance concept was evaluated on a scale of 1–3: (1) used the term ‘partial artemisinin resistance’ and explained it appropriately, (2) did not mention the term ‘partial artemisinin resistance’ but explained the concept correctly, (3) did not mention the term ‘partial artemisinin resistance’ and did not explain the concept.

Additionally, if the concept ‘partial artemisinin resistance’ was transmitted, the specific sections (abstract, introduction, methods, results, discussion) were identified.

## 3 Results

**Fig. 1:**
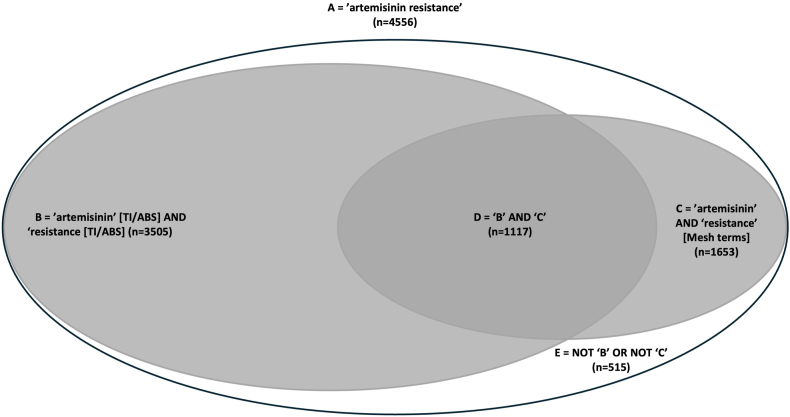
Venn Diagram of PubMed search results The diagram represents the relationship of articles retrieved by text search and MeSH terms. About a quarter of the hits (Set D) include both MeSH terms and the text terms ‘artemisinin’ AND ‘resistance’. Group E articles have a weaker relationship with the subject, as the search terms were found in other PubMed fields (see methods for detailed search description). All articles in ‘B’ OR ‘C’ (both grey sets, n = 4041) were used for further study. A: Total number of articles retrieved with the search term ‘artemisinin resistance’. B: Articles with the terms ‘artemisinin’ and ‘resistance’ in the title or abstract. C: Articles indexed with the MeSH terms ‘drug resistance’ and ‘artemisinins’. D: Articles that include both the text terms in the title/abstract and the MeSH terms (B ∩ C). E: Articles that do not include the specific text terms in the title/abstract nor the MeSH terms.

A PubMed search using the terms ‘artemisinin resistance’ retrieved 4556 articles. Of these, 3505 articles included variants of ‘artemisinin’ and ‘resistance’ in their title or abstract, while 1653 articles were only indexed with the MeSH terms ‘drug resistance’ and ‘artemisinins’ (Fig. 1, Table 2). Combining searches for words in the title and abstract (tiab) and MeSH terms search methods, a total of 4041 unique articles were identified (set E, Table 2). Only 281 (7 %) contained the words ‘partial’ or ‘delayed’ in their title or abstract (set G, Table 2), typically within the context of ‘partial artemisinin resistance’ or ‘delayed parasite clearance’, as revealed by a three- or four-word cluster analysis (Supplementary Material). This left 3760 articles (93 %) that did not include ‘partial’ or ‘delayed’ in their titles or abstracts (set J, Table 2). This was further confirmed through corpus analysis, which identified only two unrelated instances of the term ‘partial’ in a three-word cluster.

**Table 2:** PubMed search hits for ‘artemisinin resistance’.

Search strategy	Set^a^	Hits (n)
‘artemisinin resistance’ [all fields] in PubMed – default search	A	4556
All word variants of ‘artemisinin’ AND ‘resistance’ in title or abstract [TI/AB]	B	3505
‘artemisinin’ AND ‘drug resistance’ [MeSH terms]	C	1653
Set ‘B’ AND set ‘C’ (word search and MeSH terms) - overlap	D = B ∩ C	1117
Set ‘B’ OR ‘C’ (word search and MeSH terms) - combined	E = (B ∪ C) ⊆A	4041
Hits which do not have ‘B’ OR ‘C’ – search terms in other fields	F = A ∩ ¬ (B ∪ C)	515
Set ‘E’ AND (‘partial’ OR ‘delayed’ in title or abstract [TI/AB])	G = E ∩ P	281
Set ‘B’ AND (‘partial’ OR ‘delayed’ in title or abstract [TI/AB])	H = B ∩ P	267
Set ‘C’ AND (‘partial’ OR ‘delayed’ in title or abstract [TI/AB])	I = C ∩ P	143
Set ‘E’ NOT (‘G’ OR ‘H’ OR ‘I’) – countercheck for the absence of words ‘partial’ OR ‘delayed’	J = E ∩ ¬ (G ∪ H ∪ I)	3760

This table shows the number of hits for each search strategy in PubMed. Set A represents the default search strategy (in all PubMed data elements (fields) - see Medline Data Elements https://www.nlm.nih.gov/bsd/mms/medlineelements.html for details). See methods for detailed description.

a Schematic presentation of hits in set theory (see also Fig. 1): ∩ = AND, ∪ = OR, ⊆ = subset, ¬ = NOT.

**Fig. 2:**
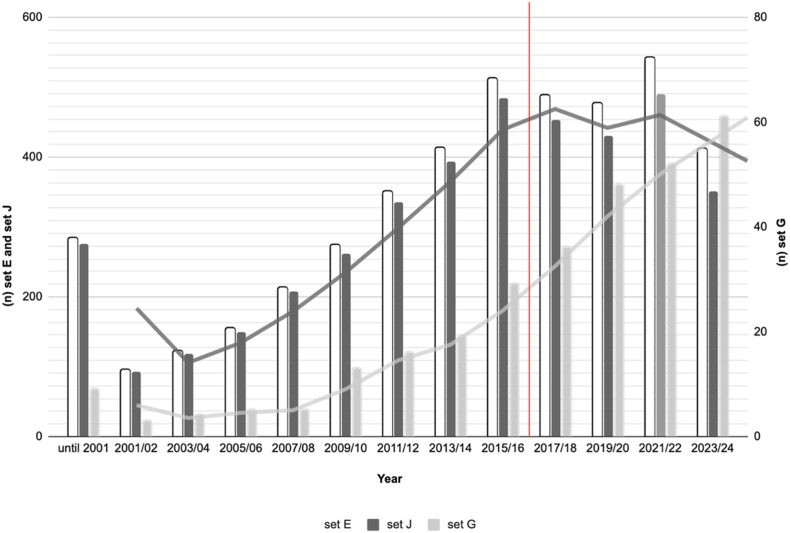
Frequency of PubMed search hits (two-year intervals) PubMed search hits per two-year interval (see methods for detailed description). The red line indicates the introduction of the term ‘partial artemisinin resistance’ by WHO. There has been a steady increase in the number of publications over the years. Only about 10 % of the retrieved articles (n = 197) since 2017 include the terms ‘partial’ or ‘delayed’ (set G), reflecting adherence to the new WHO terminology. See Table 1 for a description of sets. Set E: Open bars for ‘artemisinin resistance’ represent hits for all searches with MeSH terms and word variants (primary Y-axis, left). Set J: Dark grey bars for ‘artemisinin resistance’ without ‘partial’ or ‘delayed’. The dark grey line represents the trend line (moving average). Set G: Light grey bars for ‘artemisinin resistance’ with ‘partial’ or ‘delayed’. The light grey line represents the trend line (moving average) (secondary Y-axis, right).

When the WHO introduced the term ‘partial artemisinin resistance’ in 2018 (indicated by the red line in Fig. 2), there was no significant change in the number of publications that did not use this term in their titles or abstracts. However, there was a small proportional increase in publications that included this term to explain the underlying concept, although this increase was based on a very small absolute number (n = 197), representing only 10 % of the retrieved articles since 2018 (Fig. 2).

For further analysis, subset ‘J’ (n = 3760 articles) was used, which excludes any article with ‘partial’ or ‘delayed’ in their titles or abstracts (Table 2). A search for ‘artemisinin resistance’ in the titles or abstracts in the WoS database resulted in 3409 articles, with 1390 published post-2018 and having a DOI. Filtering these results to include only those in PubMed subset ‘J’, with more than 10 citations, and confirming the absence of ‘partial’ or ‘delayed’ in their titles or abstracts, 461 articles were identified (Table 3).

**Table 3:** This table summarizes 460 articles from Web of Science (WoS) and PubMed, published after 2017, each having a DOI and more than 10 citations and Web of Science search results for 460 hits.

Description	(n)	Comment
Article type (total)	(460)	WoS classification of article type, grouped into four categories.
- Original article	336	
- Review	113	
- Editorial	8	
- Letter	3	
WoS journal category (total)	(460)	Others contain many areas including: Pharmacology & Pharmacy, Multidisciplinary Sciences, Biochemistry & Molecular Biology, Chemistry, Medicinal, Public, Environmental & Occupational Health, Plant Sciences, Biotechnology, Genetics, Biophysics, and Cell Biology.
- Infect Dis	32	
- Infect Dis, Microbiol/Parasitol	22	
- Med, Infect Dis, Trop Med, Parasitol	89	
- Med	49	
- Microbiol/Parasitol	68	
- Other	200	
Citations (total)	(460)	Eight papers had citations above 200: 223, 246, 263, 279, 303, 315, 381, 547, respectively
− 10-20	211	
− 21-30	105	
− 31-40	49	
− 41-50	36	
− 51-100	40	
− 101-150	7	
− 151-200	4	
- >200	8	
Top 10 Journals (groups) (total)	(460)	Total of 187 different journals. Journals grouped by publisher. Others contain many areas (n) including frequently published journals such as ACS Journals (9), Molecules (9), J Med Chem (7), Int J Parasitol-Drug (7), Trends Journals (7), and New Engl J Med (5), as well as journals from different fields such as Phytomedicine (3), Science (3), Antioxidants-Basel (2), Cell Host Microbe (2), Genome Med (1), Microorganisms (1), and Vaccine (1).
- Malaria J	70	
- Plos Journals	22	
- Antimicrob Agents Ch	17	
- Lancet Journals	16	
- Frontiers Journals	15	
- Eur J Med Chem	14	
- Nature Journals	13	
- Current Journals	12	
- Sci Rep-Uk	12	
- J Clin Infect & Clin Infect Dis	10	
- Others	242	

It categorizes the articles by type (original, review, editorial, letter), WoS journal classifications, and citation ranges. It also highlights journals with articles having >10 citations (note: journals from the same publisher are grouped together).

**Fig. 3:**
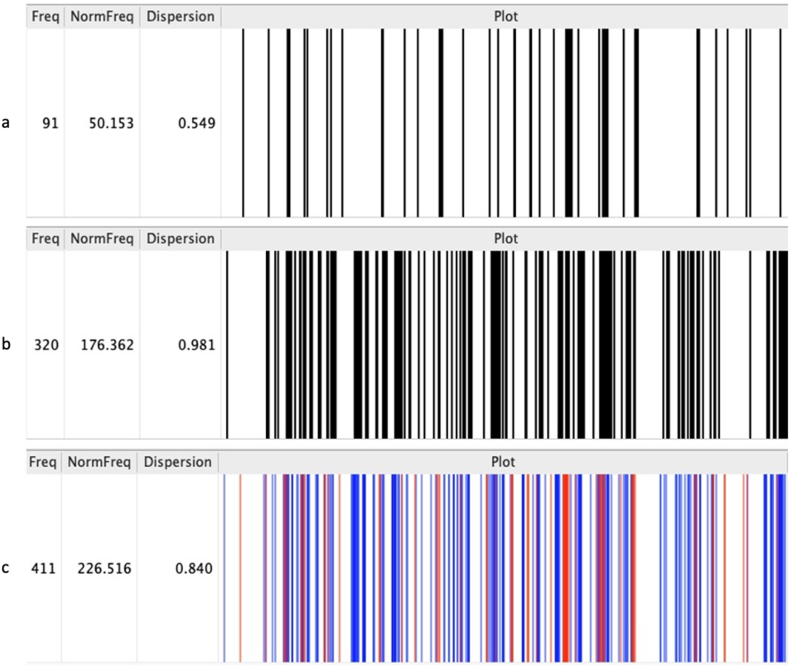
Distribution of terms ‘partial’ and ‘delayed’ in key publications (n = 198) AntConc distribution analysis of the terms ‘partial’ (panel a, n = 91) and ‘delayed’ (panel b, n = 320) in the corpus of 198 PDF. The panel c shows an overlay of both terms (‘partial’: red, ‘delayed’: blue), totaling 411 occurrences. The normalized frequency indicates term appearance per set token count. Juilland's D measures dispersion, where 0 indicates maximum clustering (terms appearing in a few documents) and 1 indicates even distribution (terms evenly spread across documents).

Of the 209 selected articles (59 with over 50 citations, and 150 randomly selected from those cited fewer than 50), 198 PDFs were successfully retrieved and analyzed using AntConc, creating a TXT corpus of 1,814,445 tokens. ‘Resistance’ appeared 7364 times, ‘artemisinin resistance’ 1503 times, and ‘resistance to artemisinin’ 147 times. The full term used by the WHO, ‘partial artemisinin resistance’, was found only 12 times, and ‘partial resistance’ appeared 23 times, while ‘delayed’, often used to describe the WHO's concept of partial resistance, occurred 320 times. A three-word cluster analysis of ‘delayed’ retrieved 226 instances directly associated with the WHO concept of partial resistance, with the distribution of these instances being homogeneous (Fig. 3).

Manual review excluded 37 unrelated articles, leaving 161 articles. Among these, ‘partial’ was rarely used: 97 % of basic research, 88 % of clinical research, and 93 % of epidemiological studies did not include the term ‘partial’ (Table 4). ‘Delayed’ was more frequently mentioned in basic research articles (71 %) than in clinical (44 %) or epidemiological studies (30 %).

**Table 4:** Concept of ‘Partial Artemisinin Resistance’ in selected full-text articles (n = 161).

	Basic Research (n = 87)		Clinical Studies (n = 34)		Epidemiological Studies (n = 40)	
Interest to general clinical reader/public						

• Very likely	2	(2 %)	30	(88 %)	6	(15 %)
• Perhaps likely	19	(22 %)	4	(12 %)	23	(58 %)
• Not likely	66	(76 %)	0	(0 %)	11	(28 %)

Use the term ‘partial’						

• Yes	3	(2 %)	4	(12 %)	3	(8 %)
• No	84	(97 %)	30	(88 %)	37	(93 %)

Use the term ‘delayed’						

• Yes	62	(71 %)	15	(44 %)	12	(30 %)
• No	21	(24 %)	19	(56 %)	24	(60 %)

Transmission of ‘Partial Resistance’ concept						

• Well explained	10	(11 %)	3	(9 %)	4	(10 %)
• Mentioned	18	(21 %)	19	(56 %)	12	(30 %)
• Not mentioned	59	(68 %)	12	(35 %)	24	(60 %)

Sections explaining ‘Partial Artemisinin Resistance’						

• Abstract	1	(1 %)	0	(0 %)	0	(0 %)
• Introduction	24	(27 %)	12	(35 %)	12	(30 %)
• Results	2	(2 %)	2	(6 %)	0	(0 %)
• Methods	0	(0 %)	0	(0 %)	2	(5 %)
• Discussion	1	(1 %)	6	(18 %)	3	(7 %)
• Conclusion	0	(0 %)	0	(0 %)	1	(0 %)
• Not mentioned	57	(65 %)	12	(35 %)	24	(60 %)
• Other	5	(6 %)	4	(12 %)	2	(5 %)

This table summarizes the verification of the concept of ‘partial artemisinin resistance’ across basic research, clinical studies, and epidemiological studies, focusing on the interest to clinical readers, the use of key terms, and the sections where the term was explained.

Inaccurate communication in health sciences: the case of ‘partial artemisinin resistance’ for the treatment of malaria.

Most basic research (68 %) and epidemiological studies (60 %) did not mention or explain the concept of ‘partial artemisinin resistance’, while 55 % of clinical studies addressed it.

Regarding the section in which the term ‘partial artemisinin resistance’ was explained, most publications addressed it in the introduction (69 %). Only one publication explained it in the abstract, ten addressed it in the discussion (15 %), and six explained it in the results or methods sections (9 %). Additionally, six publications, which did not follow a conventional structure, explained the term elsewhere in the text.

## 4 Discussion

‘Partial artemisinin resistance’ is indeed a significant public health concern [23], as it has been linked to clinical failures in ACTs [17]. While these failures are often attributed to resistance to the partner drug [17], partial artemisinin resistance may represent the initial step toward full resistance, where artemisinin itself or the ACT could become clinically ineffective [32].

The term ‘partial artemisinin resistance’ was introduced only a decade after the first reports of ‘artemisinin resistance’ [20]. This 10-year lag in establishing accurate nomenclature likely contributed to the solid establishment and widespread use of inaccurate terminology ‘artemisinin resistance’ in the scientific community. However, accurate and precise use of scientific terminology is crucial in health sciences to prevent misunderstandings that can have serious consequences [33], [34], [35]. The case of ‘artemisinin resistance’ is an example of a controversial terminology [[21], [22], [23], [24],^26^], which could be considered inaccurate and potentially lead to miscommunication and even inappropriate clinical decisions.

In clinical microbiology, pathogens are generally classified using a binary system of ‘susceptible’ or ‘resistant’, with an intermediate category labelled as ‘intermediate susceptibility’ or ‘reduced susceptibility’ — not as any form of ‘initial resistance’ [35,36]. This classification is essential for guiding everyday clinical decisions: ‘susceptible’ indicates that the pathogen is likely to respond to standard treatment, ‘intermediate’ or ‘reduced susceptibility’ suggests a possible response at higher doses or in specific body sites, and ‘resistant’ denotes that the pathogen is unlikely to respond, requiring alternative therapies [35,36]. This system is consistently applied across bacteria, fungi, and usually parasites, including malaria-causing *Plasmodium* species [37,38]. For example, for antimalarial drugs like chloroquine and sulfadoxine-pyrimethamine, parasites are considered ‘resistant’ only when there is a significant loss of efficacy leading to treatment failure [39]. Usually, in clinical practice, in the field of antimicrobial resistance the terms ‘partial’ or ‘initial’ resistance are not used; instead, any decrease in susceptibility that does not result in clinical failure is categorised under ‘susceptible, increased exposure’, as clearly stated by the European Committee on Antimicrobial Susceptibility Testing (EUCAST) [2,35]. By contrast, the use of ‘artemisinin resistance’ without qualifiers rather seems to imply (full) resistance, which can lead to misinterpretation and inappropriate clinical decisions. This deviation from standard antimicrobial resistance terminology risks causing clinicians to wrongly perceive artemisinin as ineffective, even though it remains clinically effective in most cases [13].

Our findings revealed that 93 % of articles did not use the more accurate term of ‘partial artemisinin resistance’ or explain it, risking confusion. However, within the 7 % that used or explained the term correctly, most included it in the introduction, with one even defining it in the abstract, sections that tend to capture the most reader attention [40]. By focusing our study on highly cited publications (>10 citations) across all years since 2018, we may had excluded more recent publications, particularly from 2023 to 2024 that did not have the chance to be cited as frequently. However, we believe the selected sample is representative of the broader literature on this topic and does not influence the overall results or conclusions.

It is critical to ensure that terms are used correctly in scientific publications, especially given the hierarchical flow of information from specialized scientific literature to the general media and, ultimately, the public [[29], [30], [31]]. The use of correct terms in scientific publications helps guarantee that the information is accurately understood by non-specialists, including policymakers and clinicians.

## 5 Conclusion

In conclusion, our study highlights the need for the timely and consistent implementation of precise terminology in scientific communication. Adopting the term ‘partial artemisinin resistance’ or ‘reduced susceptibility to artemisinin’ would align with established clinical microbiology practices and better reflect current drug efficacy. This consistency is critical for ensuring that effective treatments are not prematurely abandoned and that patients receive the most appropriate care.

## CRediT authorship contribution statement

**T. Hanscheid:** Writing – review & editing, Writing – original draft, Validation, Methodology, Formal analysis, Conceptualization. **Sara M. Mahomed:** Writing – review & editing, Writing – original draft, Validation, Formal analysis. **Maria Rebelo:** Writing – review & editing, Writing – original draft, Validation, Formal analysis. **Susana Oliveira Henriques:** Writing – review & editing, Methodology. **Martin P. Grobusch:** Writing – review & editing, Writing – original draft, Methodology, Conceptualization.

## Ethical approval

Not required.

## Declaration of generative AI and AI-assisted technologies in the writing process

We have used AI (OpenAI's GPT, version 4.0), to enhance the language and readability of our manuscript. This application of AI technology did not replace any essential tasks such as producing scientific insights, analysing and interpreting data, or drawing scientific conclusions. We disclose this writing assistance in accordance with journal guidelines.

## Funding source

None.

## Declaration of competing interest

The authors declare that they have no known competing financial interests or personal relationships that could have appeared to influence the work reported in this paper.
