# Supplementary material for: Inaccurate communication in health sciences: The case of ‘partial artemisinin resistance’ for the treatment of malaria
Source: New Microbes New Infect. 2024 Nov 30;62:101544. doi: 10.1016/j.nmni.2024.101544 (PMC11667688; doi:10.1016/j.nmni.2024.101544)
Supplement: Multimedia component 1 [file mmc1.docx]

*Supplementary Material*

**Inaccurate communication in health sciences: the case of ‘partial artemisinin resistance’ for the treatment of malaria**

# S1. Artificial Intelligence Analysis Tools and Programs used

*S1.1 GPT-4*

GPT is an advanced language model developed by OpenAI. It is capable of understanding and generating human-like text, making it useful for tasks such as generating search terms, analyzing text, and providing detailed assessments of complex concepts. In this study, we employed GPT (version 4.0) to generate search terms for the systematic review. The rationale for using GPT stems from its ability to efficiently process vast amounts of data and offer comprehensive linguistic variations for keywords, ensuring more exhaustive and precise search results. By incorporating GPT into the search process, we were able to: (1) **improve search precision:** GPT's ability to generate synonyms and related phrases minimized the risk of missing relevant literature that might have used slightly different terminology; (2) **increase search coverage:** GPT helped identify emerging terms that may not have been included in traditional keyword searches; (3) **efficiency in term variation generation:** the tool reduced the manual effort typically required to compile all possible variants of search terms (e.g., ‘artemisinins’, ‘artemisinine’, ‘resistance’, ‘delayed clearance’). This ensured that our search strategy was both comprehensive and inclusive of all potentially relevant studies.

*S1.2 AntConc*

AntConc (version 4.2.4, released on September 25, 2023), developed by Laurence Anthony, is a freeware corpus analysis toolkit designed for analyzing large collections of text. This tool facilitates various tasks such as identifying word frequencies, examining linguistic patterns, and extracting features from the text corpus. In this study, AntConc was used to conduct a detailed corpus analysis of the extracted titles and abstracts from the retrieved hits, focusing on the terminology used in the literature.

In corpus analysis, particularly with AntConc, a corpus is treated as a single, continuous text made up of multiple documents. If you want to analyze, for example, 200 papers, you would combine them into one corpus. This allows AntConc to process all the documents as a unified dataset, enabling you to extract patterns, frequencies, and other linguistic features across the entire collection. Essentially, the tool analyses the whole set of texts as one cohesive unit, rather than treating each document separately.

The documents were converted into a merged TXT file and analyzed using AntConc to identify the presence and distribution of key terms like ‘resistance’ and ‘artemisinin resistance’. To assess how well the WHO concept of ‘partial artemisinin resistance’, defined as delayed parasite clearance, is conveyed in the literature, the analysis also included the terms ‘partial’ and ‘delayed’.

Data from the analysis provided insights into the frequency and distribution of these specific search terms across the entire corpus. Frequency is the number of times a search term appears in the text. For example, the term ‘partial’ appeared 91 times in the corpus. Dispersion indicates how evenly the search term is distributed throughout the text. A dispersion value close to 1 signifies an even distribution, while a value closer to 0 suggests clustering in specific sections. For example, the dispersion value for ‘delayed’ was 0.981, indicating an even distribution. In contrast, the term 'partial' had a dispersion of 0.549, indicating a more clustered distribution, suggesting that its usage was concentrated in specific sections of the text rather than being spread evenly.

**S2. Tables**

**Suppl. Table 1 – Exact search terms**

| Search Terms |
| --- |
| - Artemisinin Variants:   - "artemisinin"[Title/Abstract]   - "artemisinine"[Title/Abstract]   - "artemisinins"[Title/Abstract]   - "artemisinin's"[Title/Abstract] - Resistance Variants:   - "resist"[Title/Abstract]   - "resistance"[Title/Abstract]   - "resistances"[Title/Abstract]   - "resistant"[Title/Abstract]   - "resistants"[Title/Abstract]   - "resisted"[Title/Abstract]   - "resistence"[Title/Abstract]   - "resistences"[Title/Abstract]   - "resistent"[Title/Abstract]   - "resistibility"[Title/Abstract]   - "resisting"[Title/Abstract]   - "resistive"[Title/Abstract]   - "resistively"[Title/Abstract]   - "resistivities"[Title/Abstract]   - "resistivity"[Title/Abstract]   - "resists"[Title/Abstract] |

This table summarizes the exact search terms used for Pubmed and Web of Science (WoS) research articles.

**Suppl. Table 2 - Criteria for Verification of the Concept of ‘Partial Artemisinin Resistance’ in Selected Full-Text Articles**

| Criteria for Verification of the Concept of ‘Partial Artemisinin Resistance’ | |
| --- | --- |
| Classification of publications | - Basic research - Epidemiological - Clinical studies |
| Likelihood of interest | - **(1) Very Likely:** articles such as trials on drug efficacy or clinically relevant epidemiology data - **(2)** **Perhaps Likely:** articles covering sub-aspects like methods of partial resistance detection or clinically relevant comparisons with other drugs - **(3)** **Unlikely**: highly specialized molecular or genetic details, or where artemisinin resistance was only relevant in a remote context of the paper |
| Transmission of the concept | - **(1)** Used the term ‘partial artemisinin resistance’ and explained it appropriately - **(2)** Did not mention the term ‘partial artemisinin resistance’ but explained the concept correctly - **(3)** Did not mention the term ‘partial artemisinin resistance’ and did not explain the concept |

This table summarizes the criteria for verification of the concept of ‘partial artemisinin resistance’ in the 161 selected full-text articles
